# Stroke Prevention in Atrial Fibrillation: Understanding the New Oral Anticoagulants Dabigatran, Rivaroxaban, and Apixaban

**DOI:** 10.1155/2012/108983

**Published:** 2012-09-10

**Authors:** Tan Ru San, Mark Yan Yee Chan, Teo Wee Siong, Tang Kok Foo, Ng Kheng Siang, Sze Huar Lee, Ching Chi Keong

**Affiliations:** ^1^Department of Cardiology, National Heart Centre Singapore, 17 Third Hospital Avenue, Singapore 168752; ^2^Duke-NUS Graduate Medical School, 8 College Road, Singapore 169857; ^3^Department of Cardiology, National University Heart Centre, 5 Lower Kent Ridge Road, Singapore 119074; ^4^Mount Elizabeth Medical Centre, 3 Mount Elizabeth No. 14-10, Singapore 228510; ^5^Mount Elizabeth Medical Centre, 3 Mount Elizabeth No. 14-01, Singapore 228510; ^6^Gleneagles Medical Centre, 6 Napier Road No. 03-04, Singapore 25499; ^7^Department of Neurology, National Neuroscience Institute, Tan Tock Seng Hospital, 11 Jalan Tan Tock Seng, Singapore 308433

## Abstract

Unlike vitamin K antagonists (VKAs), the new oral anticoagulants (NOACs)—direct thrombin inhibitor, dabigatran, and direct activated factor X inhibitors, rivaroxaban, and apixaban—do not require routine INR monitoring. Compared to VKAs, they possess relatively rapid onset of action and short halflives, but vary in relative degrees of renal excretion as well as interaction with p-glycoprotein membrane transporters and liver cytochrome P450 metabolic enzymes. Recent completed phase III trials comparing NOACs with VKAs for stroke prevention in atrial fibrillation (AF)—the RE-LY, ROCKET AF, and ARISTOTLE trials—demonstrated at least noninferior efficacy, largely driven by significant reductions in haemorrhagic stroke. Major and nonmajor clinically relevant bleeding rates were acceptable compared to VKAs. Of note, the NOACs caused significantly less intracranial haemorrhagic events compared to VKAs, the mechanisms of which are not completely clear. With convenient fixed-dose administration, the NOACs facilitate anticoagulant management in AF in the community, which has hitherto been grossly underutilised. Guidelines should evolve towards simplicity in anticipation of greater use of NOACs among primary care physicians. At the same time, the need for caution with their use in patients with severely impaired renal function should be emphasised.

## 1. Introduction

Atrial fibrillation (AF) increases the risk of embolic stroke. Anticoagulation with vitamin K antagonists (VKAs), dose-adjusted to achieve a target international normalised ratio (INR) range of 2.0-3.0, significantly reduces stroke risk meta-analysis revealed a significant stroke risk reduction of 64% (CI, 49% to 74%) compared to placebo [1]—with acceptable rates of bleeding complications [1, 2]; but is limited by inherent problems. These problems include a narrow drug therapeutic index, wide variations in metabolism, and numerous food and drug interactions [3]. Hence, there is a need for regular monitoring of INR. Aspirin has limited efficacy for stroke prevention in AF. A meta-analysis showed a significant stroke risk reduction of 37% (CI, 23% to 48%) with VKA compared to aspirin and a trend towards stroke risk reduction of 19% (CI, −1% to 35%) with aspirin compared to placebo, which just missed statistical significance [1]. Furthermore the risk of bleeding on aspirin therapy is not inconsiderable [4, 5]. Combination therapy with aspirin and clopidogrel in the Atrial Fibrillation Clopidogrel Trial with Irbesartan for Prevention of Vascular Events (ACTIVE-A) trial was better than aspirin alone for prevention of vascular events but was associated with increased bleeding events [6]. However, this combination failed to prevent vascular events compared to standard VKA treatment [7].

The stroke prevention using oral thrombin inhibitor in atrial fibrillation (SPORTIF AF) trials, which compared fixed-dose direct thrombin inhibitor ximelagatran with an optimally dose adjusted VKA, provided the first clinical support for the feasibility of chronic anticoagulation without INR monitoring [8–10]. However, ximelagatran was subsequently withdrawn due to safety concerns [11, 12]. Recent trials of new oral anticoagulants (NOACs) have demonstrated efficacy for stroke prevention in AF and good safety profiles, without detectable hepatotoxicity signals [13–16]. They promise a new era of anticoagulation management in AF [17].

## 2. The New Oral Anticoagulants

Dabigatran, an oral direct thrombin inhibitor, and rivaroxaban and apixaban, both oral direct activated factor X inhibitors, exhibited favourable pharmacodynamics and predictable pharmacokinetic profiles in early phase trials, making them candidate alternatives to VKAs [22–25]. Of these, dabigatran [13], rivaroxaban [14], and apixaban [15, 16] have completed phase III clinical trial programmes for stroke prevention in AF, with the first two already approved by key regulatory agencies worldwide.

## 3. Pharmacodynamics and Pharmacokinetics

Dabigatran inhibits thrombin directly, and potentially the various downstream actions of thrombin [26]. The highly selective direct inhibitors of factor Xa, rivaroxaban and apixaban, inhibit factor Xa activation of prothrombin to thrombin with limited effects outside the coagulation cascade [27]. Compared to thrombin, factor Xa is more thrombogenic [27, 28] and activates clotting over a wider concentration range, with a shallow dose-response curve that implies a wider therapeutic window [27]. Coagulation times as measured by conventional prothrombin time (PT) and activated partial thromboplastin time (aPTT) are prolonged by the factor Xa antagonists, and dabigatran, respectively [29–31]. Unlike INR for VKAs, prolongation of PT and aPTT can neither be used to gauge adequacy of anticoagulation nor titrate the dose of the NOAC.

**Figure 1:**
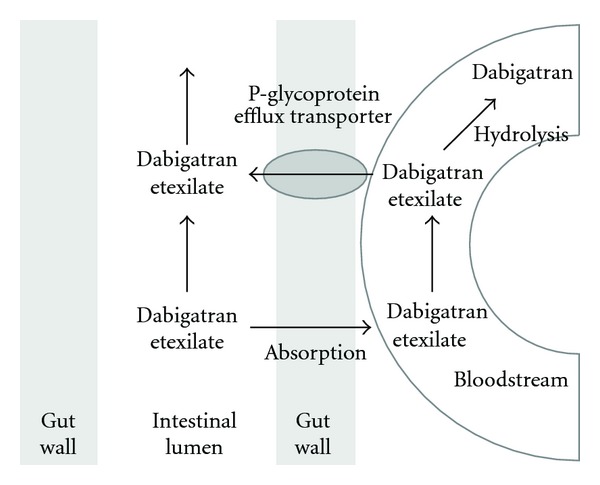
Dabigatran etexilate: a p-gp substrate. Using energy from adenosine triphosphate, p-gp receptors in the intestinal wall actively transport molecules across the epithelial monolayer. Because of its low bioavailability, dabigatran etexilate, with its moderate affinity for the p-gp receptor, is sensitive to the actions of p-gp efflux at the intestinal wall. Once absorbed into the intestinal bloodstream, dabigatran etexilate is hydrolysed by plasma esterases to its active principle, dabigatran. The latter is no longer a substrate for p-gp efflux.

Key pharmacokinetic characteristics of dabigatran, rivaroxaban and apixaban are summarised in Table 1 [18–20]. Dabigatran etexilate, a prodrug, is hydrolysed by plasma esterases into its active principle, dabigatran, after intestinal absorption (Figure 1). Unlike rivaroxaban and apixaban, dabigatran etexilate absorption is slow and acid sensitive. Hence, a coating of dabigatran etexilate is applied onto a tartaric acid core to form tiny pellets contained within gel capsules. The higher rates of dyspepsia observed with dabigatran (versus warfarin) may be due to the tartaric acid core in the dabigatran formulation [32]. Concomitant use of proton pump inhibitors reduces bioavailability of dabigatran by about 20%, which is neither deemed clinically significant nor merits dose adjustment [32]. 

Compared to VKAs, the NOACs have rapid onset of action and short halflives. For stroke prevention in AF, dabigatran and apixaban are administered twice daily, and rivaroxaban once daily. The choice of once-daily dosing for rivaroxaban was based on phase II data demonstrating efficacy with such a regimen [33] and the observation that rivaroxaban's anticoagulant activity manifest as inhibition of prothrombinase-induced thrombin generation, persists up to 24 hours after an administered dose [34].

Dabigatran is predominantly excreted by the renal route. In contrast, only approximately 33% and 25% of active rivaroxaban and apixaban, respectively, are excreted unchanged in the urine with the rest being excreted by the biliary route or converted by liver enzymes, such as cytochrome P450, into inactive metabolites [18–20]. 

Rivaroxaban, apixaban and dabigatran etexilate (but not dabigatran) are substrates of p-glycoprotein (p-gp), a ubiquitous transmembrane receptor found in the intestinal wall, as well as various other locations in the body, including the blood-brain barrier. P-gp actively transports molecules with diverse conformations, including drugs, across tissue monolayers [35]. Compared to rivaroxaban and apixaban, oral dabigatran etexilate's slow intestinal absorption renders it relatively more sensitive to p-gp efflux in the gut (Figure 1). Drugs that inhibit (e.g., ketoconazole, verapamil, and amiodarone) or induce (rifampicin, St John's wort) p-gp can potentiate or attenuate dabigatran's anticoagulant effect, respectively [18]. Rivaroxaban possesses good bioavailability and requires concomitant strong inhibition of intestinal wall p-gp and liver CYP3A4 by drugs like ketoconazole or ritonavir for a clinically significant increase in blood concentrations [19].

Dabigatran is less highly protein bound in the blood compared to rivaroxaban and apixaban and is dialysable, with about 50% removed in 4 hours [36]. The usefulness of dialysis in the setting of overdose or bleeding has been reported in anecdotal cases but has yet to be systematically and rigorously studied.

There is no specific antidote currently available for NOACs although preclinical studies using a recombinant monoclonal antibody targeting dabigatran or reconstructed factor Xa protein hold promise [37, 38]. Nonspecific pro-coagulants, such as nonactivated and activated prothrombin complex concentrate (PCC) and recombinant factor VIIa have been advocated for management of major bleeding or rapid reversal for emergency-invasive procedures [39]. Initial *in vivo* testing of PCC administration in healthy volunteers exposed to therapeutic doses of rivaroxaban and dabigatran demonstrated rapid normalisation of coagulation times and clotting parameters in the former but not the latter [40]. However, a distinction should be drawn between reversal of coagulation parameters and actual reduction of bleeding [41]. In another study of mice given supratherapeutic doses of dabigatran, PCC reduced both bleeding time and size of intracerebral haematoma [42]. The use of PCC in the clinical setting has not been studied, and, until more results are available, their application must be balanced against their potential powerful prothrombotic tendencies. 

## 4. The Phase III Clinical Trials: Trial Design

Efficacy and safety of NOACs versus VKAs for stroke prevention in patients with AF were evaluated in three trials: randomized evaluation of long-term anticoagulation therapy (RE-LY), rivaroxaban once daily oral direct factor Xa inhibition compared with vitamin K antagonism for prevention of stroke and embolism trial in atrial fibrillation (ROCKET AF), and apixaban for reduction in stroke and other thromboembolic events in atrial fibrillation (ARISTOTLE) [13–15]. These three studies constitute the largest prospective VKA-controlled trials of anticoagulation for stroke prevention in patients with AF with median followup of about 2 years each (Table 2). The apixaban versus acetylsalicylic acid to prevent strokes (AVERROES) trial, in which apixaban was demonstrated to be superior for stroke prevention compared to aspirin in patients with AF who are not suitable for a VKA [16], shall not be further discussed in this review. 

Patients were randomised in double-blind, double-dummy fashion in ROCKET AF and ARISTOTLE to either active treatment or VKA. In RE-LY, prospective open-label blinded endpoint evaluation (PROBE) trial design obviated the logistical difficulties of mounting a large-scale international double-blind VKA-controlled trial. With blinded endpoint evaluation used to minimise bias, subjects were randomised in a ratio of 1 : 1 : 1 to receive dabigatran 110 mg twice daily, dabigatran 150 mg twice daily, or open-label VKA. The two doses of dabigatran tested were double blinded. While both study designs have advantages and limitations, it is worth noting the impact of trial design on outcomes [43]. In the SPORTIF AF trials, primary efficacy outcome event rates for ximelagatran were similar in both SPORTIF III (open-label) and V (double-blind) trials, but event rates on the VKAs were disparate, higher in the former and lower in the latter [8–10].

The common primary objective was to demonstrate that NOACs were noninferior to VKAs for the primary efficacy endpoint of stroke or systemic embolism. Noninferiority margins, at <1.5, were relatively conservative [13–15]. Primary efficacy analyses were intention-to-treat (ITT) in RE-LY and ARISTOTLE, and per protocol on-treatment in ROCKET AF. While intention-to-treat is the most appropriate for a superiority hypothesis, consensus guidelines from the consolidated standards of reporting trials group (CONSORT) consider on-treatment analysis more appropriate for demonstration of noninferiority [44]. 

The most robust result in a noninferiority trial would be to demonstrate consistency of the on-treatment and intention-to-treat analyses, but this is not always achievable in practice [7–9, 13]. Both on-treatment and intention-to-treat analyses are subjected to different types of biases. On-treatment analysis avoids biases resulting from differential adherence to study drug in a noninferiority trial. This is especially pertinent where treatment is continuous and demanding, and withdrawal rates expected to be high. Discontinuation of study drug reduces the contrast between the study groups making it more difficult to show a difference; that is, it becomes easier to show noninferiority [44]. However, a different type of bias is introduced because on-treatment analysis may not preserve the integrity of the randomised comparison. Thus, the validity of the on-treatment analysis shall require that subjects excluded from the analysis in both arms of the trial be, at the minimum, balanced in terms of their withdrawal rates, reasons for withdrawal, and baseline characteristics. These criteria are more likely to be met with a trial design where subjects and investigators are strictly blinded to the treatment assignation than with an open-label trial design.

Severe renal impairment, defined as creatinine clearance (CrCl) calculated by the Cockcroft-Gault formula of <30 mL/min in RE-LY and ROCKET AF and <25 mL/min in ARISTOTLE, excluded participation in all three trials. This guarded against drug overexposure in subjects with advanced age and low body weight, as age, body weight, gender, and serum creatinine value were incorporated into the equation [45]. In ROCKET AF, slightly more than one-fifth of trial subjects had moderate renal impairment (CrCl 30–49 mL/min) and were given a lower rivaroxaban dose of 15 mg once daily instead of the primary dose of 20 mg once daily [14]. In ARISTOTLE, about 15% of subjects had moderate renal impairment based on estimated CrCl, but only 4.7% and 4.4% in the active and control groups, respectively, received the renal dose of apixaban 2.5 mg twice daily instead of the primary dose of 5 mg twice daily, after fulfilling two or more of the following criteria: age ≥ 80 years, weight ≤ 60 kg, or Cr ≥ 1.5 mg/dL [15]. Although it is estimated that dabigatran concentration would increase about threefold in patients with moderate renal impairment compared to those with normal kidney function [46], dabigatran dose was not stratified by CrCl in RE-LY. 

## 5. The Phase III Clinical Trials: Results

Baseline characteristics of the study populations in the trials are summarised in Table 3. All three trials recruited sizable numbers of female, VKA naïve, elderly, and renal-impaired patients. ROCKET AF recruited trial patients with unprecedentedly high risk: mean CHADS2 score of 3.5 [14] versus 2.1 in RE-LY [13] and ARISTOTLE [15]. More than half of ROCKET AF subjects had prior stroke, and nearly two-thirds had heart failure at baseline. Heart failure episodes destabilise INR and make tight INR control challenging [47], which may be one of the possible explanations that may account for the lower mean time in therapeutic range (TTR) in ROCKET AF (55%) compared to RE-LY (64%) and ARISTOTLE (62%) [13–15].

Withdrawal rates at end of study exceeded 20% in ROCKET AF, ARISTOTLE and the dabigatran arms of RE-LY [13–15]. There was a significant difference in study drug discontinuation rates between dabigatran-(21%) and VKA-treated (17%) subjects in RE-LY, which may be explained by the open-label design, as well as higher rates of dyspepsia with dabigatran (possibly attributable to the drug's acid-containing formulation) [13, 48]. While temporary disruption of study drug for clinical indications was allowed in all three trials, prolonged disruption obligated permanent discontinuation of study drug in ROCKET AF, in line with the overall trial design which specified a per protocol on-treatment primary analysis plan. This resulted in nearly 120 days' difference between median durations of study drug exposure and study followup. Consequently, ITT analysis that does not take into consideration protocol compliance may not be ideal for demonstration of noninferiority compared to VKAs.

### 5.1. Efficacy Outcomes

Although expedient, cross-trial comparisons of outcome results should be tempered with the knowledge that in the case of NOACs, direct comparisons are hazardous due to the differences in drug classes and drug types, trial designs and analytic approaches, as well as the risk profiles of the study populations. The best method is to compare drugs in large-scale head-to-head randomised trials, but this is unlikely to happen soon. 

For the primary efficacy outcome of stroke and systemic embolism, the event rates in the VKA controls were 1.71, 2.2, and 1.60 per 100 patient-years in RE-LY, ROCKET AF, and ARISTOTLE, respectively [14, 15, 21].  Table 4 summarises the efficacy outcomes in the active treatment groups, expressed as relative risk reductions (RRRs) or increments of event rates, compared to VKA-controlled populations in the trials [13–15, 21]. There were significant RRRs for dabigatran 150 mg and apixaban that maintained their significance when tested for superiority [15, 21]. In the dabigatran 110 mg group, *P* value was significant at <0.05 for noninferiority testing, but not for superiority [21]. With rivaroxaban, *P* values were <0.05 for noninferiority and superiority with prespecified on-treatment analyses, which censored events occurring up to 48 hours after the study drug was permanently withdrawn, but superiority was not seen with sensitivity analysis based on ITT, which counted all events up to a common date of site notification of end of study treatment [14]. 

Haemorrhagic strokes, included as stroke outcome events, were significantly reduced with all NOACs [14, 15, 21]. Ischaemic stroke events were significantly reduced with dabigatran 150 mg but increased, albeit insignificantly, with dabigatran 110 mg [21]. Cardiovascular and all-cause mortality were numerically reduced with all three drugs [14, 15, 21].

Myocardial infarction (MI) event rates in the VKA control arms were very low at 0.64, 1.11 and 0.61 per 100 patient-years in RE-LY, ROCKET AF, and ARISTOTLE, respectively [14, 15, 21]. Although not statistically significant, there were numerically fewer incidents of MI with the factor Xa inhibitors and more with dabigatran, when compared with the VKA arms. Notably, results from the recent phase III anti-Xa therapy to lower cardiovascular events in addition to standard therapy in subjects with acute coronary syndrome-thrombolysis in myocardial infarction 51 (ATLAS ACS 2-TIMI 51) trial showed that, compared to placebo, the addition of rivaroxaban 2.5 mg or 5.0 mg twice daily (one quarter and one half of the daily dose in ROCKET AF) to dual antiplatelet therapy significantly reduced the risk of the composite endpoint of MI, stroke, or death from cardiovascular causes in patients with a recent acute coronary syndrome [49].

In the initial RE-LY publication, MI events with dabigatran 150 mg twice daily were significantly higher than with a VKA [13]. After including incident silent MI events, the significant increase in MI was no longer nominally significant. Importantly, the main finding of the RE-LY trial remained unaltered [21]. A recent meta-analysis of randomised studies of dabigatran for diverse indications showed a small signal for MI with dabigatran [50]. However, the weight of evidence shall have to lie chiefly with RE-LY, the largest trial for dabigatran, which ultimately did not demonstrate a statistically significant increase in MI risk. Scrutinising MI events in isolation may be unrepresentative. A RE-LY subanalysis found myocardial ischaemic events (composite of MI, unstable angina, cardiac arrest, and cardiac death) to be numerically, albeit not statistically significantly, lower among subjects randomised to dabigatran compared to the VKA [51]. 

In RE-LY, benefits of dabigatran treatment over the VKA were independent of stroke risk stratification (i.e., CHADS2 score) [52] prior VKA use [53], prior stroke status [54], and quality of INR control [55]. Reported subanalyses of ROCKET AF confirm the same for rivaroxaban with regard to INR control [14], prior stroke status [56], prior MI status [57], and renal insufficiency [58]. 

### 5.2. Safety Outcomes

All three trials adopted International Society of Thrombosis and Haemostasis bleeding criteria but differed slightly in the finer details of the exact definitions for major bleeding, as well as the choice of the primary safety endpoint: major bleeding in RE-LY and ARISTOTLE, and the composite of major and nonmajor clinically relevant bleeding in ROCKET AF. Among VKA-treated patients in RE-LY, ROCKET AF and ARISTOTLE, major bleeding event rates were 3.57, 3.45 and 3.09 per 100 patient-years, and intracranial haemorrhage (ICH) event rates were 0.76, 0.74, and 0.80 per 100 patient-years, respectively [14, 15, 21].  Table 5 summarises the safety outcomes in the active treatment groups, expressed as RRR or increments of event rates, compared to VKA-controlled populations. Major bleeds, defined as bleeding associated with a haemoglobin drop of 2 g/dL or more, transfusion requirement of 2 units or more, critical organ bleed, or fatal bleed, were significantly reduced with dabigatran 110 mg and apixaban [15, 21]. Gastrointestinal bleeds were increased with dabigatran 150 mg and rivaroxaban, but these did not translate into potentially lethal bleeds for both doses of dabigatran or fatal bleeding with rivaroxaban, the event rates of which were significantly reduced [14, 21].

There were significant reductions in ICH that mirror the significant RRRs observed in haemorrhagic stroke events. Taken together, they suggest a brain-protective effect for these NOACs, the exact mechanism of which remains to be elucidated. A more stable anticoagulant effect compared to that of a VKA does not fully explain this phenomenon, as ICH reductions were observed at all levels of centre TTR [55]. Cell-surface tissue factor (TF), which is found in high concentrations in the brain, may offer an explanation [59]. TF form TF-VIIa complexes that initiate coagulation. TF-VIIa complexes are suppressed by VKAs, which block vitamin K-dependent carboxylation of factor VII, but not by NOACs with their more highly selective targets. 

Dabigatran is speculated to possess limited ability to penetrate the blood-brain barrier [54, 60]. In animal models, rivaroxaban and apixaban are found in much lower concentrations in the brain compared to plasma [61–63]. P-gp, and possibly other yet to be identified cotransporters, may play a role in this (rivaroxaban and apixaban, but not active dabigatran, are p-gp substrates) [63]. P-gp efflux transporters in the blood-brain barrier provide protection against entry of potential noxious endogenous and exogenous compounds, and have been implicated in development of resistance to oncological and microbiological therapeutic agents [64]. In experimental p-gp double knockout mice, brain-to-blood concentration ratios of rivaroxaban 15 and 60 minutes after oral administration were 1.6 and 3.2 times higher, respectively, compared to wild-type mice [63]. This implies that efflux of rivaroxaban from the brain to the blood may be modulated by p-gp action and inhibition.

## 6. Postmarketing Surveillance of NOACs

Rapidly increasing use of dabigatran, the first NOAC to receive approval for prevention of stroke in AF, and increased awareness about the drug have led to higher than usual reporting of bleeding events related to the drug. The bleeding events, including fatal ones, occurred up to several months after initiation of treatment. Some have been linked to use of dabigatran in elderly patients with severe renal impairment, a group that is known to have increased bleeding risk [59]. Safety advisories have been issued by several jurisdictions [65–67], and physician prescribers reminded about contraindications to the drug as well as the need for vigilance in monitoring of bleeding complications and renal function deterioration.

The reports of increased bleeding in the postmarketing surveillance phase should be interpreted in context. While several hundred fatal events have occurred, it is impossible to interpret these data without information concerning the total number of patients treated with dabigatran as well as information concerning the expected event rates if these same patients had been treated with warfarin.Currently, the postmarketing experience of bleeding events associated with dabigatran has not altered its overall benefit-risk profile [67, 68]. 

It may be appropriate to distinguish between dabigatran, which is predominantly renally cleared, and the factor Xa inhibitors which are one-quarter to one-third renally cleared. Of note, rivaroxaban has been approved by North American regulatory agencies in patients with a creatinine clearance as low as 15 mL/min. Dabigatran has been approved by the FDA for patients with a creatinine clearance of 15–30 mL/min, albeit with a lower dose that was not tested in the trials [69].

Even though NOACs were designed to be used without routine coagulation monitoring, the concern over bleeding has spurred interest in coagulation testing of NOACs, especially in specific circumstances such as surgery, overdose, and bleeding. Commonly available coagulation assays such as PT and aPTT time are not suitable for routine coagulation monitoring of the NOACs, but may provide qualitative information concerning the presence or absence of the drug, especially in the surgical setting. Thrombin time and Ecarin clotting time have been shown to correlate well with plasma dabigatran concentration, but are not routinely available outside of research laboratories. Thrombin clotting time tests calibrated to dabigatran, and factor Xa assays calibrated to rivaroxaban have been developed. However, outside of the specific circumstances alluded to above, the use of these tests for dose adjustment of NOACs remains untested and contentious. 

## 7. Discussion 

Despite their efficacy for preventing stroke in patients with AF, VKAs are grossly underused, especially in the elderly: a population with rapidly increasing AF prevalence [70] that actually stands to benefit the most from VKA treatment. Major obstacles to VKA therapy include the cost and inconvenience of obligatory INR monitoring with VKAs, as well as the risk of bleeding (especially ICH). 

Guidelines recommend VKAs for higher risk AF patients on the basis of estimated efficacy benefits versus the bleeding risk with treatment [71, 72]. Older AF stroke risk assessment tools possess modest sensitivity and specificity [73]. The newer CHA2DS2-VASc score [74] accurately identifies patients at very low risk for stroke (score of 0 confers 0.8% or less stroke risk at one year) [74, 75] and stratifies the rest for antithrombotic treatment, with VKAs being preferred over antiplatelet therapy [71, 72]. High bleeding risk, as determined for instance by HAS-BLED score ≥ 3 [76], justifies caution in initiating antithrombotic therapy.

NOACs fundamentally alter the above-treatment paradigm. With their efficacy for stroke prevention at least equal to VKAs and convenient fixed-dose administration, acceptability among physicians and patients should increase. The use of antiplatelet treatment as expedient, albeit not very efficacious, substitutes a VKA for stroke prevention in AF will consequently decrease. Furthermore, the striking reductions in ICH with these NOACs compared to VKAs make these drugs eminently suitable for use in patients at high risk of ICH, a fact best exemplified by ROCKET AF, which recruited patients at high risk of stroke and bleeding. Bleeding scores such as HAS-BLED have been validated with VKAs but not with NOACs. As such, they may not predict similar degrees of ICH risk, which are significantly attenuated with NOACs. While extracranial bleeds are still troubling, they do not appear to increase the rates of fatal or potentially fatal bleeds. 

NOACs are becoming the new standard for anticoagulation in AF [77]. As the burden of AF in the community greatly exceeds the capacity of specialist care, these NOACs open up opportunities for primary care physicians to initiate and maintain anticoagulation treatment in the wider, hitherto undertreated, group of eligible patients with AF. Guidelines must commensurately evolve and be made simpler to reflect this new possibility. Perhaps, in the near future, except for male patients younger than 65 years without any other risk factors (i.e., CHA2DS2-VASc score 0), all other patients with AF should be anticoagulated, preferably with a NOAC.

VKAs will still continue to have a role for patients with AF using mechanical prosthetic valves (where the optimal INR target range is higher than that for patients with AF), significant valve disease and end-stage renal failure, all of whom have been excluded from participation in the AF trials to date.

## 8. Conclusion

NOACs are at least equal to VKAs for stroke prevention in AF. Dabigatran 150 mg twice daily and apixaban showed superiority on ITT analyses, the acknowledged gold standard for assessing superiority, while rivaroxaban has superior efficacy based on on-treatment analysis. The different study designs make direct comparison of the efficacy effect sizes of the different NOACs versus VKAs unreliable. Major and nonmajor clinically relevant bleeding rates with NOACs are acceptable compared to VKAs. There were significant reductions in intracranial bleeds observed for all the new drugs, the mechanism of which, including the role of membrane transport molecules such as p-gp at the blood-brain barrier, remains to be fully elucidated. While dabigatran 150 mg twice daily and rivaroxaban appear to cause more gastrointestinal bleeding than VKAs, life-threatening or fatal bleeds with the respective NOACs were in fact significantly reduced. Of the three NOACs, apixaban demonstrated reduced bleeding (major and/or nonmajor clinically relevant bleeds) compared to a VKA.

ROCKET AF studied a significantly higher-risk group of AF patients compared to RE-LY and ARISTOTLE, representing the population most difficult to treat who are at increased risk of both thromboembolic events and bleeds. This makes direct comparison of trial results misleading. Once-a-day dosing differentiates rivaroxaban from dabigatran and apixaban and may enhance patient acceptance and compliance with treatment. 

Not only are NOACs alternatives to VKAs for stroke prevention in patients with AF, but also they appear to hit the sweet spot of both improved efficacy and safety. Guidelines should evolve towards simplicity in anticipation of greater use of NOACs for primary care treatment of AF in the community.

## Figures and Tables

**Table 1:** Pharmacokinetic properties of dabigatran, rivaroxaban, and apixaban.

	Dabigatran [18]	Rivaroxaban [19]	Apixaban [20]
Prodrug	Dabigatran etexilate	No	No
Bioavailability	6.5%, pH sensitive	>80%	>50%
Time to peak, h	0.5–2	2–4	3-4 h
Plasma halflife, h	12–14	9–13	8–15
Renal elimination of active drug	85%	33%	27%
Liver CYP3A4 substrate	No	Yes	Yes
P-glycoprotein substrate	Dabigatran etexilate, but not dabigatran	Yes	Yes
Protein binding	34-35%	92–95%	87%
Dialysability	Yes	Not expected	Unlikely

**Table 2:** RE-LY, ROCKET AF, and ARISTOTLE trial design and conduct.

	RE-LY [13]	ROCKET AF [14]	ARISTOTLE [15]
Patient number	18,113	14,264	18,201
Median followup, years	2.0	1.9	1.8
Trial design	PROBE*	Double blind	Double blind
Study drug	Dabigatran	Rivaroxaban	Apixaban
Study drug dose(s)	110 mg or 150 mg BD	20 mg OD	5 mg BD
Renal dose	None	15 mg OD	2.5 mg BD
Comparator	Open-label warfarin	Warfarin	Warfarin
Primary objective	Noninferior efficacy	Non-inferior efficacy	Non-inferior efficacy
Noninferiority margin(s)	1.46	1.46	1.44; 1.38 (log scale)
Primary efficacy analysis	Intention-to-treat	On treatment	Intention-to-treat

*PROBE: prospective open-label blinded endpoint evaluation; BD: twice daily; OD: once daily.

**Table 3:** Baseline demographics in RE-LY, ROCKET AF, and ARISTOTLE.

	RE-LY [13]			ROCKET AF [14]		ARISTOTLE [15]	
	Dabigatran 110 mg BD	Dabigatran 150 mg BD	Warfarin	Rivaroxaban	Warfarin	Apixaban	Warfarin
	*n* = 6015	*n* = 6076	*n* = 6022	*n* = 7131	*n* = 7133	*n* = 9120	*n* = 9081
Female, %	35.7	36.8	36.7	39.7	39.7	35.5	35.0
Prior VKA use, %	50.1	50.2	48.6	62.3	62.5	57.1	57.2
Age, years^†^	71.4	71.5	71.6	73	73	70	70
CrCl < 50 mL/min, %		19.4 (for whole study)		22.4	23.2	15.0	15.2
HF/low LVEF^††^, %	32.3	31.8	31.9	62.6	62.3	35.5	35.4
Hypertension, %	78.8	78.9	78.9	90.3	90.8	87.3	87.6
Diabetes, %	23.4	23.1	23.4	40.4	39.5	25.0	24.9
Prior stroke/TIA, %	19.9	20.3	19.8	54.9^†††^	54.6^†††^	19.2	19.7
Prior MI, %	16.8	16.9	16.1	16.6	18.0	14.5	13.9
CHADS_2_ mean	2.1	2.2	2.1	3.5	3.5	2.1	2.1

BD: twice daily; CrCl: creatinine clearance; HF: heart failure; LVEF: left ventricular ejection fraction, MI: myocardial infarction; TIA: transient ischaemic attack; VKA: vitamin K antagonist.

^†^Mean in RE-LY; median for ROCKET AF and ARISTOTLE.

^††^≤ 35% in ROCKET AF; ≤40% for RE-LY and ARISTOTLE.

^†††^Includes systemic embolism.

**Table 4:** Relative risk reductions (RRRs) of efficacy outcome event rates versus VKA control groups.

	RE-LY [21]		ROCKET AF [14]	ARISTOTLE [15]
	Dabigatran 110 mg BD	Dabigatran 150 mg BD	Rivaroxaban	Apixaban
Primary outcome RRR, %	↓ 10*	↓ 35*	↓ 21*	↓ 21*
Haemorrhagic stroke RRR, %	↓ 69*	↓ 74*	↓ 41*	↓ 49*
Ischaemic/unknown stroke RRR, %	↑ 11	↓ 24*	↓ 6	↓ 8
CV death RRR, %	↓ 10	↓ 15*	↓ 11	↓ 11
All death RRR, %	↓ 9	↓ 12	↓ 15	↓ 11*
MI RRR, %	↑ 29	↑ 27	↓ 19	↓ 12

BD: twice daily; RRR: relative risk reduction.

*P < 0.05.

**Table 5:** Relative risk reductions (RRRs) of safety outcome event rates versus VKA control groups.

	RE-LY [21]		ROCKET AF [14]	ARISTOTLE [15]
	Dabigatran 110 mg	Dabigatran 150 mg	Rivaroxaban	Apixaban
Major bleed* RRR, %	↓ 20*	↓ 7	↑ 4	↓ 31*
Intracranial bleed** RRR, %	↓ 70*	↓ 59*	↓ 33*	↓ 58*
Fatal bleed RRR, %	—	—	↓ 50*	↓ 39^∗∗#^
Potentially lethal bleed RRR, %	↓ 33*	↓ 20*	—	—
Gastrointestinal bleed RRR, %	↑ 8	↑ 48*	↑ 46^∗#^	↓ 11
Major and nonmajor clinically relevant bleeds RRR, %^†^	↓ 22*	↓ 9*	↑ 3	↓ 32*

RRR: relative risk reduction.

*P < 0.05.

**In modified intention-to-treat analysis, statistical significance was not reported.

^#^Comparison was made using reported raw event frequencies, as annualised event rates were not available.
